# Efficient Cadmium-Tolerant Phosphate-Solubilizing Bacteria Loaded on Bone-Derived Biochar Effectively Alleviated Cadmium Pollution and Phosphorus Deficiency in *Brassica napus* L. Plantation

**DOI:** 10.3390/microorganisms14092072

**Published:** 2026-09-16

**Authors:** Zhilian Gong, Xiao Zhang, Zhengyan Chou, Lingshan Li, Xuejiao Chen, Yong Li, Lin Wan, Zhipeng Xie, Jinrui Zhang, Haocheng Wang, Ziyuan Qiu

**Affiliations:** 1 College of Food and Biological Engineering, Xihua University, Chengdu 610039, China; 2 Faculty of Environmental Engineering, Southwest Jiaotong University, Chengdu 610059, China

**Keywords:** CdTPSB, phosphate-solubilizing functional gene, root-cell-wall Cd retention, P deficiency, Cd-contaminated control

## Abstract

Both cadmium (Cd) contamination and phosphorus deficiency pose major constraints to sustainable crop production. This study loaded highly efficient Cd-tolerant phosphate-solubilizing bacteria (CdTPSB) onto calcium- and phosphorus-rich pig bone biochar (PBB) to construct a functional composite (MPBB), which was subsequently applied in a *Brassica napus* L. pot experiment. The results revealed that the CdTPSB possessed strong capabilities in phosphate solubilization (383.67–520.19 mg L^−1^), Cd immobilization (64.67–66.67%), IAA production (113.24–114.27 μg mL^−1^), and siderophore activity (65.02–89.08%). Batch experiments further showed that MPBB’s efficiencies in phosphate solubilization and Cd immobilization were significantly enhanced, which was putatively attributable to improved CdTPSB colonization and viability as well as to the activation of PBB functional groups. In the pot experiment, relative to the control treatment, MPBB significantly decreased the soil diethylenetriaminepentaacetic acid-extractable Cd content by 50.47% while increasing the soil labile phosphorus content by 3.32 times (*p* ≤ 0.05). This was correlated with the enrichment of Cd-immobilizing and phosphate-solubilizing bacteria, the upregulation of phosphate-solubilizing functional genes (*pqqC* and *phoD*), and enhanced soil enzyme activity. Consequently, Cd content in the shoots of *Brassica napus* L. was further decreased by 74.00%, which was correlated with increased polysaccharide content and enhanced Cd retention in root cell walls. Overall, this study highlighted MPBB’s potential as an eco-friendly Cd remediation agent and phosphate fertilizer to support the sustainable production of *Brassica napus* L.

## 1. Introduction

Phosphorus (P) is key to crop growth and fundamental physiological processes such as photosynthesis and protein synthesis [1]. To boost crop yields, mineral phosphate fertilizers have been extensively applied in agricultural systems. However, the excessive application of phosphate fertilizer results in low P use efficiency and heavy metal contamination, particularly with cadmium (Cd). In China, the Cd content in over 7% of agricultural soil exceeds the soil quality standard for agricultural land [2]. Once taken up by plants, Cd induces oxidative stress, leading to membrane lipid peroxidation, chlorophyll degradation, and the inhibition of protein synthesis [3]. *Brassica napus* L. is an essential oil crop and a widely consumed leafy vegetable [4,5]; however, it is susceptible to Cd accumulation. Therefore, the exploration of environmentally friendly fertilization strategies that can mitigate Cd pollution and P deficiency in *Brassica napus* L. cultivation is a matter of urgency.

Cadmium-tolerant phosphate-solubilizing bacteria (CdTPSB) are considered an environmentally friendly biofertilizer with the potential to alleviate Cd pollution and replace up to 50% of mineral phosphate fertilizer [6,7]. However, the practical application of CdTPSB remains constrained by low survival rates and reduced activity in soil environments, largely owing to environmental stressors and competition from indigenous microbial communities.

Biochar is widely recognized as an eco-friendly soil amendment that can immobilize heavy metals and enhance soil nutrient status. It is also an effective carrier for functional microorganisms owing to characteristics such as large specific surface area, high porosity, and abundant nutrients [8,9]. Most previous studies focused on plant-derived and functional bacteria-loaded plant-derived biochar [1,10]. However, bone-derived biochar is richer in nutrients, including calcium (Ca), P, nitrogen, and other minerals, and can theoretically replace 13–32% of the global demand for phosphate fertilizer [8,11]. Although bone-derived biochar, especially that obtained from heavily contaminated areas, might pose a risk of co-contamination with heavy metals such as Pb or As present in bone, the safety of bone-derived biochar can be ensured by strictly controlling the quality of bone feedback and using high-temperature pyrolysis to reduce heavy metal bioavailability [12]. Compared with other types of bone-derived biochar such as those from cows and chickens, pig-bone-derived biochar has higher hydroxyapatite content at 0.928 g g^−1^ [13]. With the aid of phosphate-solubilizing bacteria (PSB), more Ca and P could be released from bone-derived biochar, which is beneficial for heavy metal immobilization [7,14]. However, the performance and underlying mechanisms of CdTPSB-loaded pig-bone-derived biochar in decreasing soil bioavailable Cd content, alleviating P deficiency, and reducing Cd accumulation in crops remain poorly understood.

In this study, efficient CdTPSB were isolated from an ore district and loaded onto pig bone biochar (PBB) to construct a functional composite (MPBB). Subsequently, the composite was applied in a *Brassica napus* L. pot experiment to explore its performance and mechanisms in controlling Cd pollution and alleviating P deficiency in the *Brassica napus* L. cropping system. We hypothesized that (i) MPBB would effectively increase soil P availability and Cd immobilization by improving the colonization and viability of efficient CdTPSB, the activation of functional groups on PBB, soil enzyme activity, the soil bacterial community, and the abundance of phosphate-solubilizing genes and (ii) MPBB would further decrease the Cd content in *Brassica napus* L. shoots by enhancing root cell wall composition and Cd retention, putatively owing to the high IAA production capacity of CdTPSB and Ca release from PBB. This study provides a new strategy and comprehensive insights into the sustainable cultivation of *Brassica napus* L. through the integration of microbial functionality with engineered bone-derived biochar rich in Ca and P.

## 2. Materials and Methods

### 2.1. Biological Characteristics and Identification of CdTPSB

Two non-antagonistic CdTPSB, F9 and F1-2-3, were previously isolated from an ore district in Sichuan Province using solid NBRIP (National Botanical Research Institute’s phosphate growth) medium supplemented with 100 mg L^−1^ Cd. The Cd immobilization rate was determined following the method of Qin et al. (2023) [15]. Phosphate-solubilizing capacity was quantified in liquid NBRIP medium as described by Xie et al. (2021) [16]. The 1-aminocyclopropane-1-carboxylate (ACC) deaminase activity was determined according to the method of Penrose and Glick (2003) [17]. Siderophore activity and IAA production were determined using the chrome azurol S assay and the Salkowski reagent colorimetric assay, respectively [18]. Taxonomic identification of the strains was performed according to the method described by Wakarera et al. (2022) [19].

### 2.2. Microbial Biochar Composite Preparation and Selection

Strains F9 and F1-2-3 were mixed at a 1:1 ratio to form a functional bacterial consortium (M). To identify an optimal carrier for CdTPSB, rice husk biochar (RHB) and pig bone biochar were both prepared via pyrolysis at 500 °C for 2 h under oxygen-free conditions (heating ramp of 10 °C min^−1^ and N_2_ at a flow rate of 100 mL min^−1^), and the microbial rice husk biochar (MRHB) and microbial pig bone biochar (MPBB) were then prepared via physical adsorption [9]. The bacterial loading efficiency of MRHB and MPBB was assessed according to the method of Zhang et al. (2023) [20], and MPBB was found to exhibit superior performance (Table S1). Therefore, MPBB was selected for the subsequent experiments.

### 2.3. Batch Experiment of MPBB in Cd-Polluted Inorganic Phosphate Medium

To explore the performance and underlying mechanisms of MPBB in Cd immobilization and phosphate solubilization, batch experiments were performed by adding M (5.5 mL; 1.0 × 10^8^ CFU mL^−1^), PBB (2%), and MPBB (2%), respectively, into liquid NBRIP medium containing 200 μM Cd, according to the method described by Qi et al. (2023) [9]. During an 8-day incubation at 30 °C and 180 rpm in the dark, 5 mL samples were collected at 0, 6, 12, 24, 48, 96, and 192 h. All samples were filtered through 0.22 μm membrane filters prior to the determination of pH, soluble phosphate, and Cd^2+^ concentrations. Following incubation, the surface morphology and elemental composition of the precipitates in each treatment were characterized using scanning electron microscopy (SEM, Sigma360, Oberkochen, Germany) and energy-dispersive X-ray spectroscopy (SEM-EDS). Additionally, the surface functional groups of the original materials (PBB, MPBB, and M) and the resulting precipitates in each treatment were analyzed using Fourier-transform infrared spectroscopy (FTIR, PerkinElmer, Shelton, CT, USA). All samples were measured in the wavenumber range of 4000–400 cm^−1^, with 16 scans co-added per sample to improve the signal-to-noise ratio. Three independent biological replicates were analyzed for each treatment.

### 2.4. Pot Experiment Design

The effects of MPBB on soil P availability, soil bioavailable Cd, and Cd content in *Brassica napus* L. were investigated using a pot experiment. The soil used for the pot experiment was collected from farmland in Dayi County, Sichuan Province, and was artificially spiked with CdCl_2_ solution to reach a final Cd concentration of 2.66 mg kg^−1^, referring to the risk control standard for soil contamination of agricultural land [21]. The basic physicochemical properties of the soil are presented in Table S2. Four treatments were set up: (i) control (CK), consisting of 1 kg Cd-polluted soil with no amendment; (ii) 2% PBB; (iii) 2% MPBB; and (iv) bacterial suspension (110 mL, 1.0 × 10^8^ CFU mL^−1^). Each treatment was conducted in triplicate. Three seedlings of *Brassica napus* L. were planted in each pot and grew under natural light, with soil moisture maintained at 60% of field capacity for 50 days.

### 2.5. Rhizospheric Bacterial Community Structure and P-Related Gene Abundance

The rhizospheric bacterial community structure of *Brassica napus* L. was analyzed using high-throughput sequencing following the amplification of the V3–V4 region of 16S rDNA using the primers 338F and 806R. Quantitative PCR (qPCR) analysis of the *pqqC* and *phoD* genes was performed using the primers ALPS-F730 and ALPS-R1101 for the *phoD* gene and ApqqC-F and ARpqqC-R for the *pqqC* gene [22]. The amplicon sizes for *phoD* and *pqqC* were 373 bp and 305 bp, respectively. The amplification efficiency (104.66% for *phoD* and 101.96% for *pqqC*) was calculated based on the standard curve (slope *a* = −3.215, *R*^2^ = 0.9949 for *phoD* and slope *a* = −3.276, *R*^2^ = 0.9967 for *pqqC*).

### 2.6. Soil Biochemical Characteristics

Soil sucrase and alkaline phosphatase (ALP) activities were measured according to the method described by Qin et al. (2023) [15]. Soil P fractions were sequentially extracted as described by Sui et al. (1999) [23] and were quantitatively determined colorimetrically according to the method of Lu (2000) [24]. The concentration of diethylenetriaminepentaacetic acid-extractable Cd (DTPA-Cd) in soil, representing soil Cd bioavailability, was determined using inductively coupled plasma–optical emission spectrometry (ICP-OES) (Avio 200, Shelton, CT, USA).

### 2.7. Root Cell Wall Composition, Cd Content, and Biomass of Brassica napus L.

Fresh roots were used to determine the contents of pectin, hemicellulose (I and II), and lignin in the root cell wall, along with Cd content in both the root cell wall and the whole root. Root cell wall components were extracted using the method described in Liu et al. (2019) [25]. For lignin determination, fresh root samples were ground in 95% ethanol and then centrifuged. The precipitates were washed sequentially with 95% ethanol, ethanol:hexane (1:2, *v*/*v*), and acetone and then freeze-dried. The dry precipitates were treated sequentially with 25% acetyl bromide, 2 mol L^−1^ NaOH, and 7.5 mol L^−1^ hydroxylamine hydrochloride and then centrifuged. The absorbance of the supernatant was measured at 280 nm. For pectin and hemicellulose extraction, 75% ethanol was used for grinding fresh root samples. The precipitates after centrifugation were washed sequentially with ethanol, methanol:chloroform (1:1, *v*/*v*), and acetone and then freeze-dried. The dry precipitates were sequentially extracted with 0.5% ammonium oxalate in a boiling water bath for 1 h (for pectin), with 4% KOH for 12 h (for hemicellulose I), and with 24% KOH for 12 h (for hemicellulose II). The absorbance of all extracts was then measured at 280 nm. The cadmium content in the root cell wall, root, and shoot was determined using ICP-OES (Avio 200, Shelton, CT, USA) [26]. The Cd retention rate in the root cell wall was calculated as the ratio of the amount of the Cd in the root cell wall to that in the whole root. The translocation factors (TFs) for Cd in plants were calculated as the ratio of the Cd content in shoot to in root [27]. The biomass of the plant roots and shoots was measured using the oven-drying method.

### 2.8. Data Analysis

Prior to statistical analysis, all data were assessed for normality and homogeneity of variance. One-way analysis of variance (ANOVA) was performed, followed by the least significant difference (LSD) test using SPSS 27.0 to determine statistically significant differences among treatments (*p* ≤ 0.05, *n* = 3). Pearson correlation coefficient (PCC) analysis was performed using Origin 2022 software to assess the correlations between pH and soluble phosphorus content in the batch experiments.

## 3. Results and Discussion

### 3.1. Efficient CdTPSB Characteristics and Identification for CdTPSB 

**Figure 1:**
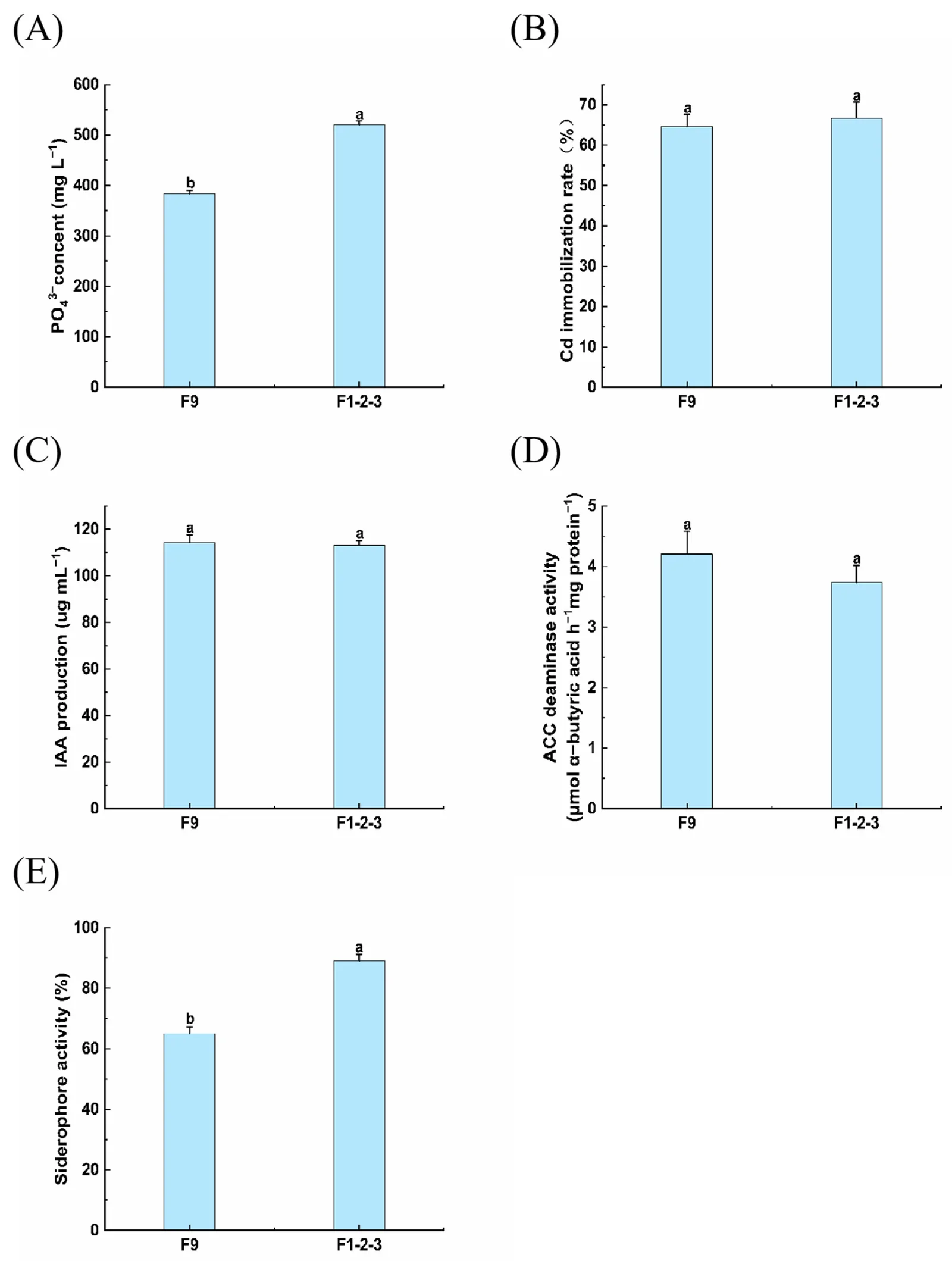
Biological characteristics of the functional bacteria, including (**A**) phosphate solubilization capacity, (**B**) Cd immobilization rate, (**C**) IAA production, (**D**) ACC deaminase activity, and (**E**) siderophore activity. Different lowercase letters above bars indicate significant differences between the data. Values represent mean ± SD of three replicates and the bars represent standard deviation (*n* = 3; *p* ≤ 0.05).

The results showed that strains F9 and F1-2-3 exhibited strong Cd immobilization capacity, with values of 64.67–66.67%, and phosphate solubilization capacity, with values of 383.67–520.19 mg L^−1^ (Figure 1A,B). These values are higher than those reported for most phosphate-solubilizing bacteria, such as *Klebsiella* sp., *Kluyvera* sp., *Acinetobacter* sp., and *K. variicola* 15-7 [15,20].

In addition, both strains exhibited high plant-growth-promoting abilities, such as IAA production (113.24–114.27 μg mL^−1^), ACC deaminase activities (3.73–4.21 μmol α-ketobutyrate h^−1^ mg^−1^ protein), and siderophore activities (65.02–89.08%) (Figure 1C–E) at values higher than those reported by Song et al. (2021) [6] and Jiang et al. (2025) [28]. These findings indicated that the two strains had strong potential as microbial remediation agents for P-deficient and Cd-polluted soil and were beneficial to crop growth. In particular, the high IAA production by the strains was beneficial to crop growth and root cell wall stability under Cd-polluted conditions [29].

**Figure 2:**
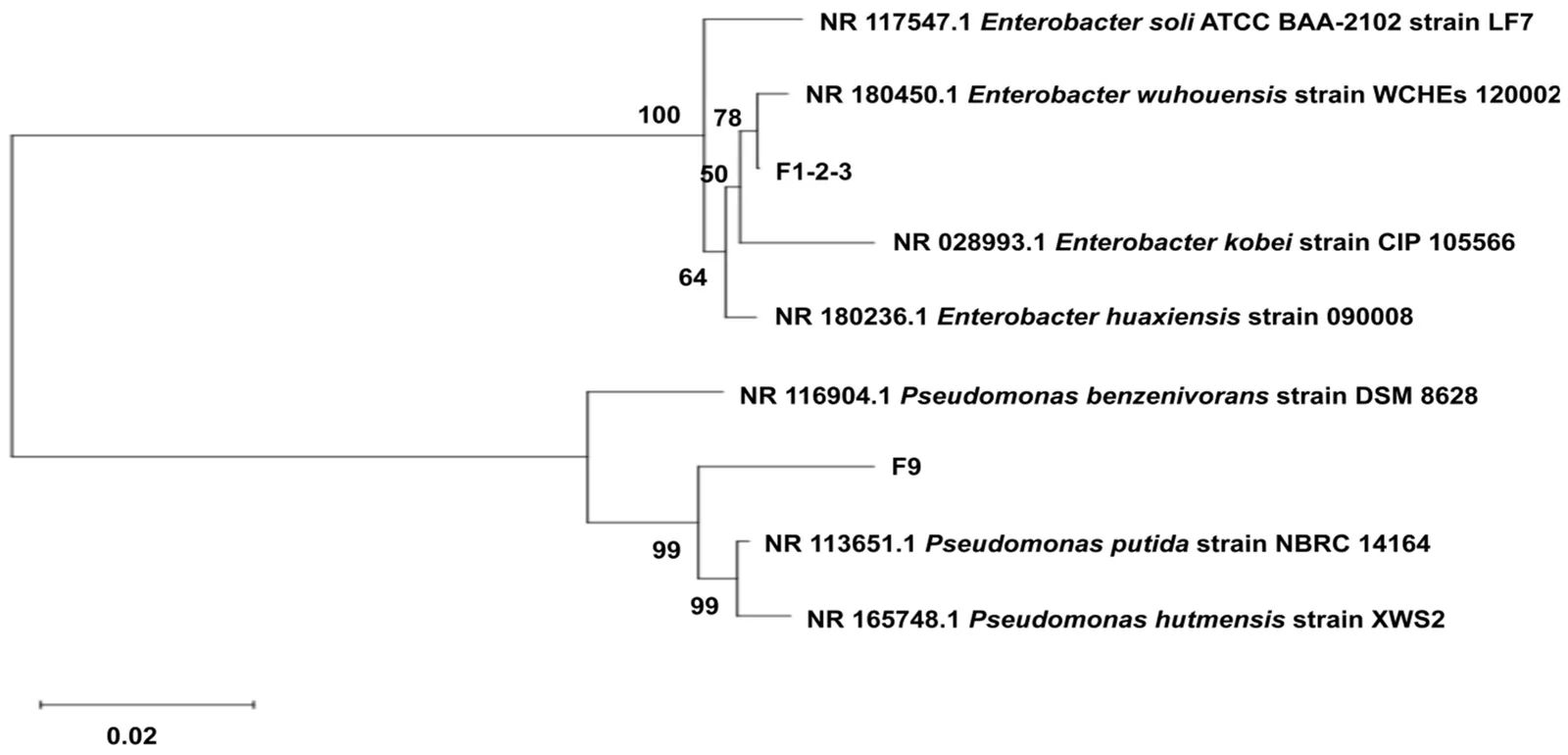
Phylogenetic tree based on 16S rDNA sequences of the functional CdTPSB.

The 16S rDNA sequences of strains F9 (OR058648.1) and F1-2-3 (PZ770282) exhibited 99.78% and 100% identity with *Pseudomonas putida* NBRC 14164 and *Enterobacter wuhouensis* WCHEs120002, respectively. Phylogenetic trees showed that F9 and F1-2-3 clustered with *P. putida* and *E. wuhouensis*, respectively (Figure 2).

### 3.2. Batch Performance of MPBB in Cd-Polluted Inorganic Phosphate Medium

**Figure 3:**
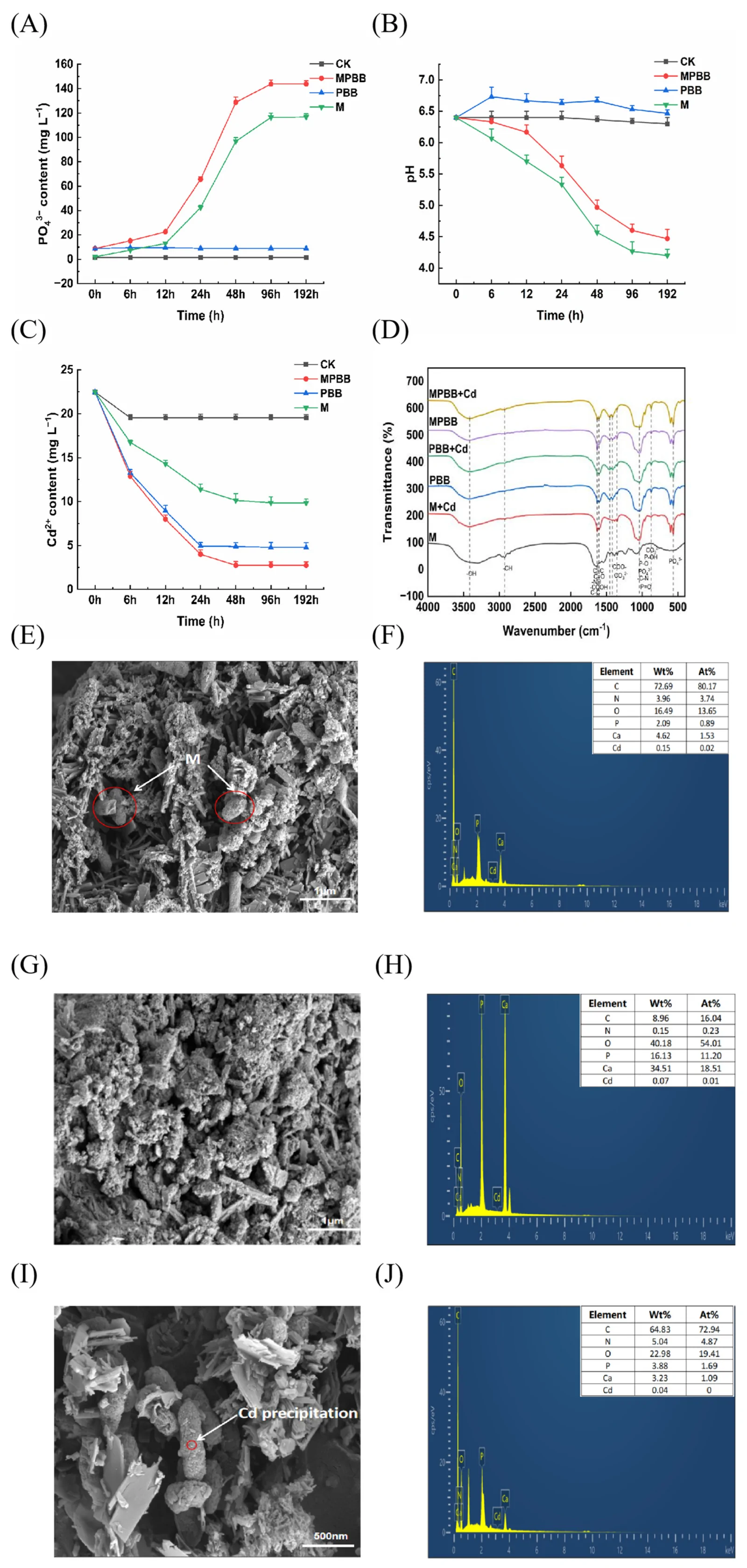
Changes in (**A**) PO_4_^3−^ content, (**B**) pH, and (**C**) Cd^2+^ content in liquid NBRIP medium in the batch test. (**D**) FTIR spectra of 3 original materials and the precipitates (MPBB + Cd, PBB + Cd, and M + Cd) after the batch experiment. SEM and EDS images for (**E**,**F**) MPBB + Cd, (**G**,**H**) PBB + Cd, and (**I**,**J**) M + Cd.

MPBB demonstrated super phosphate-solubilizing capacity. The PO_4_^3−^ concentration of liquid NBRIP medium in the MPBB and M treatments increased rapidly after 12 h incubation. At the end of incubation, the PO_4_^3−^ concentration in the MPBB treatment reached 143.75 mg L^−1^, which was significantly higher than that in the M treatment (116.67 mg L^−1^) and PBB treatment (9.04 mg L^−1^) (*p* ≤ 0.05) (Figure 3A) and also higher than that reported for PSB-loaded corn straw (56 mg L^−1^) [9]. Furthermore, the medium pH in both M and MPBB treatments decreased over the incubation period (Figure 3B), and soluble phosphate content showed a significant negative correlation with pH (*r* = −0.936, *p* ≤ 0.05). It has been reported that PSB strains solubilize phosphate by decreasing pH for organic acid production. Therefore, in this study, CdTPSB dissolved insoluble phosphate putatively through a combination of organic acid secretion and Ca^2+^ chelation [9,30].

The composite MPBB also exhibited a higher Cd immobilization rate than M and PBB alone (Figure 3C). This could putatively be attributed to the enhanced viability of CdTPSB and the richer functional groups on MPBB. Furthermore, after baseline correction and normalization, FTIR spectra indicated that, prior to the batch experiment, MPBB exhibited relatively more enhanced absorption peaks at 1631–1600 cm^−1^ (-COOH, C=C, C=O, and -NH) compared with PBB [31,32] (Figure 3D). Compared with M, MPBB exhibited more enhanced absorption peaks at 1100–873 cm^−1^ (P-OH, P-O, PO_4_^3−^, and P=O) [33] and at 566–604 cm^−1^ (PO_4_^3−^) [34] (Figure 3D). It has been suggested that MPBB may possess more active binding sites for Cd on MPBB than PBB and M [35]. After the batch experiment, the absorption peaks of C=O, C=C, -NH, and -COOH on MPBB + Cd decreased (Figure 3D), likely due to coordination and complexation between these functional groups and Cd [7,36]. In addition, the absorption peaks of P-OH, P-O, PO_4_^3−^, and P=O on MPBB + Cd showed the most pronounced broadening and intensity enhancement (Figure 3D), potentially due to strong complexation and precipitation between the P-containing groups and Cd in the MPBB treatment [37].

SEM images taken after the batch experiment showed that CdTPSB thrived in MPBB (Figure 3E). Numerous white particles were observed on the surface of the bacteria at high magnification (Figure 3E,I), and SEM-EDS analysis identified these particles as Cd-related precipitates. Furthermore, the Cd content (wt%) on the MPBB surface was 0.15, in comparison with 0.07 on PBB and 0.04 on M (Figure 3F,H,J). It could be speculated that MPBB might enhance Cd immobilization and increase soluble phosphate content.

### 3.3. Composite MPBB-Enriched Rhizospheric Beneficial Bacteria and P-Related Functional Genes

**Figure 4:**
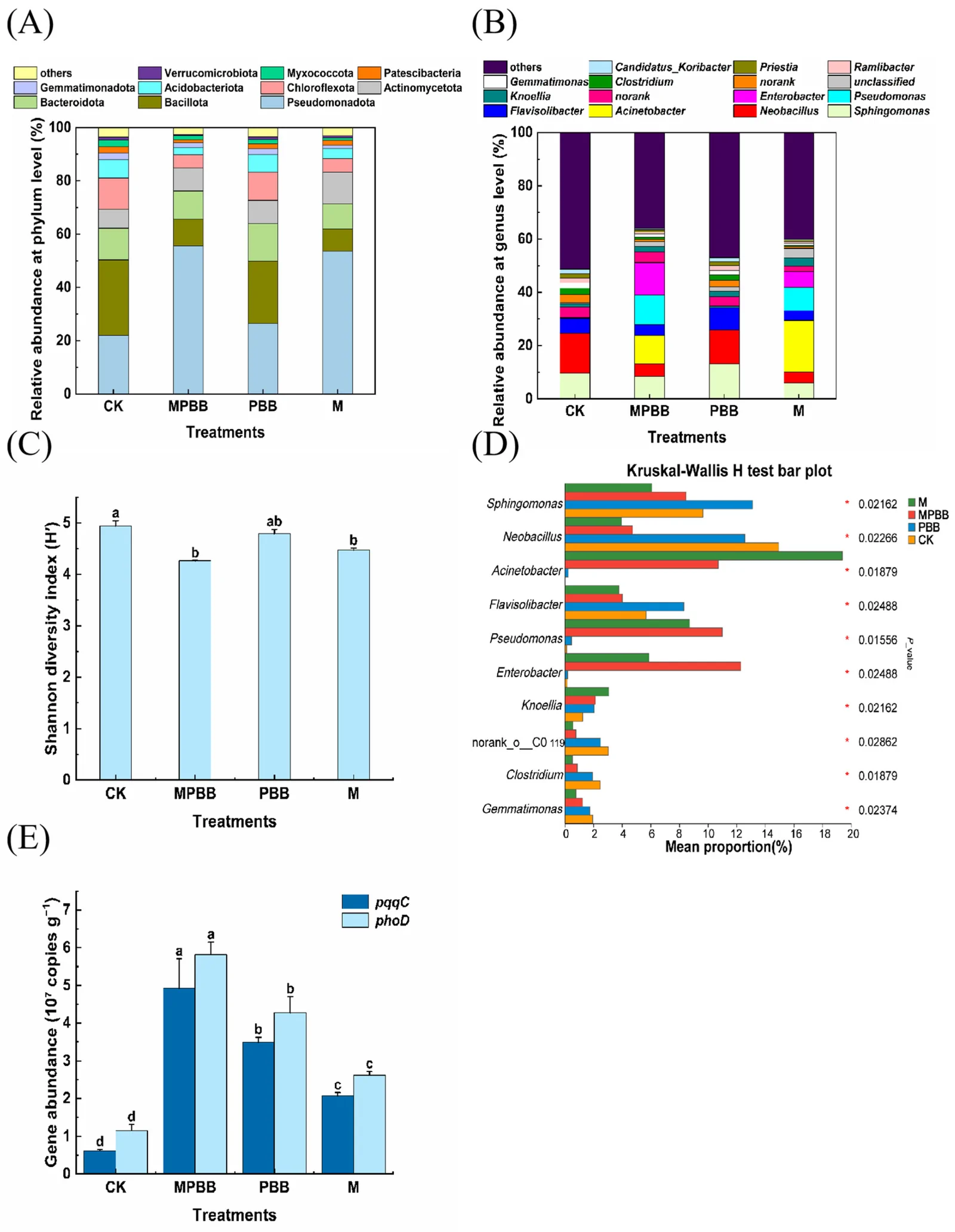
*Brassica napus* L. rhizospheric bacterial community. (**A**) Phyla relative abundance, (**B**) genera relative abundance, (**C**) Shannon diversity index (H′), (**D**) significance analysis of bacterial genus difference, and (**E**) absolute abundances of *pqqC* and *phoD* genes (mean ± SD; *n* = 3; *p* ≤ 0.05). Different lowercase letters above bars indicate significant differences between the data. Values represent mean ± SD of three replicates and the bars represent standard deviation (*n* = 3; *p* ≤ 0.05).

Microbes played a crucial role in soil nutrient cycling and heavy metal immobilization [35]. High-throughput sequencing showed that, compared with CK, the modified treatments increased the relative abundance of the dominant phyla Pseudomonadota and Actinobacteriota by 0.22–1.54 times and 0.20–0.67 times, respectively, and the dominant genera *Acinetobacter*, *Pseudomonas*, *Enterobacter*, and *Knoellia* by 9.58–843.65 times, 2.46–80.44 times, 0.22–74.24 times, and 0.63–1.43 times, respectively (Figure 4A,B). Compared with CK, the modified treatments decreased the α-diversity of the rhizospheric soil bacterial community, as reflected by the Shannon indices. The modified treatments reduced the Shannon indices by 2.86–13.63% (Figure 4C).

Notably, the MPBB treatment most effectively enriched beneficial bacterial taxa involved in P cycling and heavy metal immobilization, although it reduced the α-diversity of the rhizospheric soil bacterial community. Significance analysis at the genus level showed that the abundance of genera such as *Acinetobacter*, *Pseudomonas*, *Enterobacter*, and *Knoellia* in the MPBB treatment was significantly higher than that in the CK treatment (*p* ≤ 0.05) (Figure 4D). Furthermore, previous studies demonstrated that many members of *Pseudomonas* and *Enterobacter* exhibited strong capabilities for siderophore production, phosphate solubilization, and heavy metal immobilization [38].

Notably, this study showed that MPBB was most efficient in enriching P-related functional genes. The genes *pqqC*, encoding pyrroloquinoline quinone synthase, and *phoD*, encoding soil alkaline phosphatase, were commonly used as markers for inorganic phosphorus-solubilizing microorganisms and organic phosphorus-solubilizing microorganisms, respectively [39,40]. Compared with CK, the modified treatments significantly increased the abundance of *phoD* and *pqqC* genes by 126.49–403.69% and 238.59–703.66%, respectively (*p* ≤ 0.05) (Figure 4E), and the highest copy number of both *pqqC* and *phoD* genes was observed in the MPBB treatment.

### 3.4. Composite MPBB Effectively Decreased Soil DTPA-Cd Content and Increased P Availability

**Figure 5:**
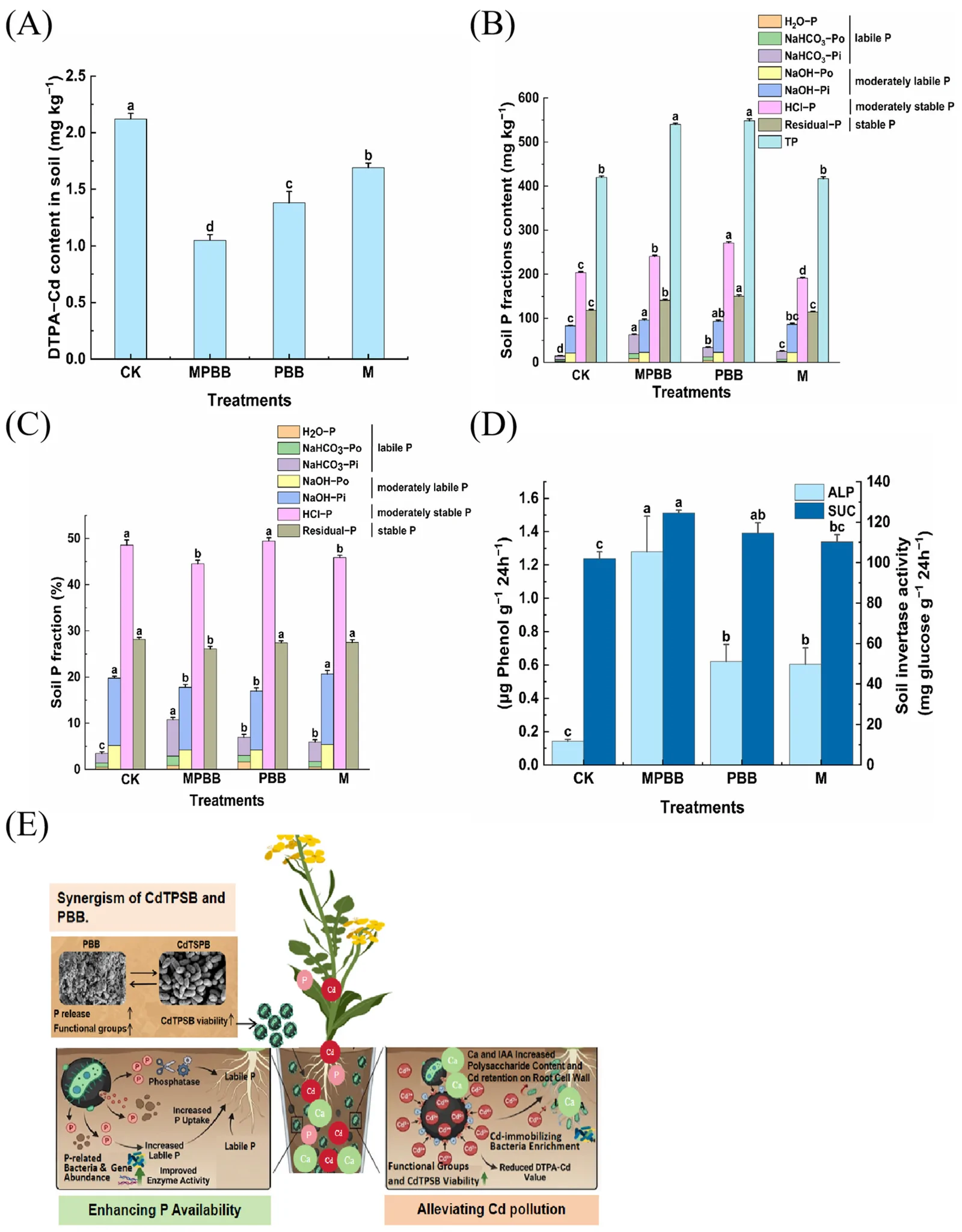
(**A**) Soil DTPA-Cd content, (**B**,**C**) content and proportion of soil P fractions, (**D**) soil sucrase and alkaline phosphatase activities, and (**E**) schematic diagram of synergisms of CdTPSB and PBB enhancing P availability and alleviating Cd pollution in planting system (mean ± SD; *n* = 3; *p* ≤ 0.05). Different lowercase letters above bars indicate significant differences between the data. Values represent mean ± SD of three replicates and the bars represent standard deviation (*n* = 3; *p* ≤ 0.05).

MPBB decreased the soil DTPA-Cd content by 50.47%, which was much more effective than CK (*p* ≤ 0.05) (Figure 5A). This was putatively attributed to the synergistic interaction between CdTPSB and PBB, which enhanced CdTPSB viability and activated oxygen- and P-containing functional groups on the MPBB surface (Figure 3D), thereby promoting MPBB to reduce Cd bioavailability via absorption, complexation, ion exchange, and precipitation processes [37,38]. It was also putatively attributed to the enrichment of heavy metal-immobilizing bacterial genera [41] such as *Pseudomonas*, *Enterobacter*, and *Acinetobacter* (Figure 4A–C).

Soil P fractions are of great importance for soil P bioavailability and soil P cycling [23]. In this study, compared with CK, MPBB most effectively increased both the content and proportion of labile P fractions (H_2_O-P, NaHCO_3_-Po, NaHCO_3_-Pi) by 3.32 times and 2.36 times (*p* ≤ 0.05) (Figure 5B,C). The content and proportion of labile P fractions in the MPBB treatment were 62.85 mg kg^−1^ and 11.62%, respectively (Figure 5B,C), which were significantly higher than those in M and PBB treatments alone (*p* ≤ 0.05) and also higher than those previously reported for PSB-loaded plant-derived biochar [20,35]. This was potentially attributed to the synergistic interaction between the efficient CdTPSB with strong phosphate-solubilizing capacities and siderophore activities (Figure 1) and PBB rich in hydroxyapatite, favorable for increasing bioavailable P content [7]. Composite MPBB solubilized hydroxyapatite and other phosphates by decreasing pH (Figure 3A,B) and chelating metal cations such as Fe^3+^, Al^3+^, and Ca^2+^ via activated functional groups such as carboxyl and hydroxyl on PBB, as well as through organic acids and siderophores secreted by CdTPSB [38]. In addition, this solubilization was attributed to the enrichment of PSB and P-related functional genes (Figure 4), as well as enhanced ALP activity (Figure 5D). Compared with CK, MPBB was most efficient in increasing sucrase activity and ALP activity, with values of 22.12% and 795.80% (Figure 5D). These comprehensive impacts of MPBB collectively promoted the conversion of stable and moderately stable P into more bioavailable fractions [42] and reduced soil DTPA-Cd content (Figure 5E).

### 3.5. Composite MPBB Increased Brassica napus L. Biomass and Decreased Shoot Cd Concentration

**Figure 6:**
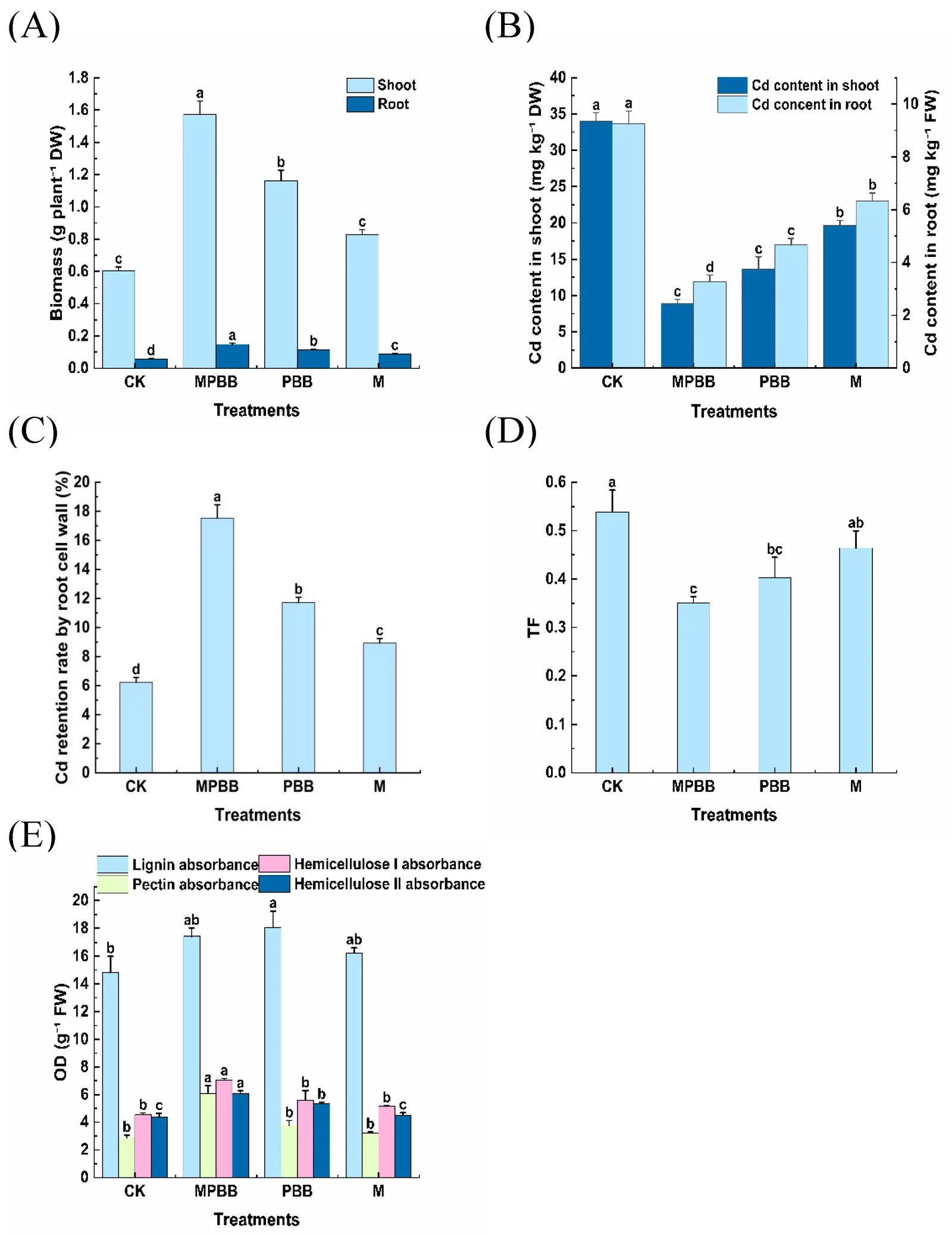
(**A**) *Brassica napus* L. biomass in shoot/root part, (**B**) Cd content in *Brassica napus* L. shoot/root part, (**C**) Cd retention rate in root cell wall, (**D**) TF for Cd in *Brassica napus* L., and (**E**) lignin and polysaccharide contents in *Brassica napus* L. root cell wall (mean ± SD; *n* = 3; *p* ≤ 0.05). Different lowercase letters above bars indicate significant differences between the data. Values represent mean ± SD of three replicates and the bars represent standard deviation (*n* = 3; *p* ≤ 0.05).

Compared with CK, the modified treatments significantly increased the biomass of *Brassica napus* L., with values of 38.13–159.92% (Figure 6A). MPBB was most efficient in promoting *Brassica napus* L. growth; this is due to CdTPSB’s high plant-growth-promoting capacities such as IAA production, siderophore activity, and phosphate solubilization, as well as the improvement in soil environmental conditions by MPBB (Figure 1, Figure 4 and Figure 5).

This study found that the modified treatments significantly enhanced the Cd retention rate in the root cell wall by 43.19–179.86% and reduced Cd contents in the shoot and root of *Brassica napus* L. by 17.66–74.00% and 31.54–64.70%, respectively, compared with CK (*p* ≤ 0.05) (Figure 6B,C). Further analysis also found that the modified treatments decreased the translocation factor of Cd, with values of 13.78–34.83% (Figure 6D).

Notably, this study innovatively found that MPBB most effectively increased the root cell wall Cd retention rate by 179.86% and decreased *Brassica napus* L. shoot Cd concentration by 74.00% for the comprehensive effect of CdTPSB functionality and engineered bone biochar (Figure 5E). The high IAA production by M and enhanced Ca^2+^ release from MPBB helped to stabilize the root cell wall and increase its polysaccharide content [29,43]. Previous reports found that Ca^2+^ was favorable for pectin stability [43] and that bacteria with high IAA production capacity could upregulate key genes involved in the biosynthesis of pectin, hemicellulose, and lignin and downregulate Cd influx transporter genes [29]. Further analysis revealed that, compared with CK, MPBB was most effective in increasing the absorbance (representing increased contents) of the extracts of pectin, hemicellulose I, and hemicellulose II in the *Brassica napus* L. root cell wall by 54.8%, 39.2%, and 10.4%, respectively (Figure 6E). The increased polysaccharides in the root cell wall in the MPBB treatment enhanced root cell wall Cd retention (Figure 6D) and inhibited heavy metal uptake and transport by binding metals via abundant functional groups such as carboxyl, hydroxyl, and aldehyde [7] (Figure 5E). Nevertheless, more sufficient evidence that high IAA production by M and enhanced Ca^2+^ release from MPBB can enhance root cell wall Cd retention needs to be obtained through future research.

In the future, field-scale research and comprehensive economic and safety analyses for MPBB will be conducted. If feedstock is obtained from low-cost food processing by-products, the high-temperature pyrolysis process could eliminate pathogens. Moreover, the implications of MPBB in soil without Cd contamination could be studied in the future.

## 4. Conclusions

In this study, efficient CdTPSB with strong capacities including phosphate solubilization (383.67–520.19 mg L^−1^), Cd immobilization (64.67–66.67%), and IAA production (113.24–114.27 μg mL^−1^) were successfully loaded onto PBB rich in P and Ca and subsequently applied in a *Brassica napus* L. pot experiment. MPBB effectively increased soil labile P content and proportion and reduced soil DTPA-Cd content, thus improving CdTPSB colonization and viability; PBB functional group activation; Ca and P release from PBB; soil enzyme activity; and soil microbial community structure, including the enrichment of phosphate-dissolving and Cd-immobilizing bacteria and P-related functional genes. MPBB further effectively decreased shoot Cd content by increasing the polysaccharide content and Cd retention in the root cell wall, potentially due to the high IAA production capacity of CdTPSB and the enhanced Ca release from PBB. These findings suggest that MPBB has strong potential as a novel and efficient bio-based Cd remediation agent and phosphate fertilizer for sustainable crop production.

## Data Availability

Data available on reasonable request.

## Supplementary Materials

The following supporting information can be downloaded at https://www.mdpi.com/article/10.3390/microorganisms14092072/s1: Table S1: The mean number of viable functional bacteria loaded on biochar; Table S2: Soil main physicochemical properties for pot experiment.

## References

[1] Liu S., Shi Y., Zhang A., Huang Y., Cao D., Lan Y. (2026). Synergistic biochar-*Bacillus* consortium enhances phosphorus availability, root architecture, and inflorescence development in greenhouse cherry tomato. Biochar.

[2] Ministry of Environmental Protection, Ministry of Land and Resources (2014). National Soil Pollution Survey Bulletin.

[3] Hu Y., He R., Mu X., Zhou Y., Li X., Wang H., Xing W., Liu D. (2025). Cadmium toxicity in plants: From transport to tolerance mechanisms. Plant Signal. Behav..

[4] Liao P., Lechon T., Harwood J.L., Scofield S. (2026). Lipid regulation in *Brassica napus*: Spatiotemporal studies to enhance our understanding. Front. Plant Sci..

[5] Shah J.A., Qin H., Zhang Z.X., Long X.X., Liu J., Raman H., Zou J. (2026). Improving pod shattering resistance in rapeseed: Insights from related *Brassica* species. Ind. Crops Prod..

[6] Song N., Wang J., Jia C., Wang F., Wang X. (2021). Biochar and *Bacillus amyloliquefaciens* NS16 can increase biomass and reduce cadmium accumulation of pakchoi explained by changes of cadmium availability, microbial biomass and community in the rhizosphere soil. Fresenius Environ. Bull..

[7] Kushwaha R., Singh R.S., Mohan D. (2023). Comparative study for sorption of arsenic on peanut shell biochar and modified peanut shell biochar. Bioresour. Technol..

[8] Ghorbani M., Azarnejad N., Brown R.W., Chadwick D.R., Loppi S., Jones D.L. (2026). Sustainable resource management with bone char—Challenges and opportunities for enhancing soil health and phosphorus stocks. Biochar.

[9] Qi W.Y., Chen H., Wang Z., Xing S.F., Song C., Yan Z., Wang S.G. (2023). Biochar-immobilized *Bacillus megaterium* enhances Cd immobilization in soil and promotes *Brassica chinensis* growth. J. Hazard. Mater..

[10] Kayoumu M., Mei Y., Liu Y., He T., Cao J., Wang Z., Zhang H.M., Duan G. (2026). Synergistic cadmium immobilization and maize growth promotion by chitosan modified biochar loaded with *Bacillus subtilis* composites: Mechanistic insights and life-cycle assessment. Bioresour. Technol..

[11] Saif I., Thakur N., Sharma M., Alalawy A.I., Jalalah M., Hassan S.H.A., Zidan N.S., Salama E. (2025). Applications of plant and animal-based biochar: Insight into environmental remediation and biofuel production. J. Water Process Eng..

[12] Vamvuka D., Dermitzakis S., Pentari D., Sfakiotakis S. (2018). Valorization of meat and bone meal through pyrolysis for soil amendment or lead adsorption from wastewaters. Food Bioprod. Process..

[13] Sawangjang B., Induvesa P., Wongrueng A., Pumas C., Wattanachira S., Rakruam P., Punyapalakul P., Takizawa S., Khan E. (2021). Evaluation of fluoride adsorption mechanism and capacity of different types of bone char. Int. J. Environ. Res. Public Health.

[14] Qu J., Wei S., Liu Y., Zhang X., Jiang Z., Tao Y., Zhang G., Zhang B., Wang L., Zhang Y. (2022). Effective lead passivation in soil by bone char/CMC-stabilized FeS composite loading with phosphate-solubilizing bacteria. J. Hazard. Mater..

[15] Qin S., Zhang H., He Y., Chen Z., Yao L., Han H. (2023). Improving radish phosphorus utilization efficiency and inhibiting Cd and Pb uptake by using heavy metal-immobilizing and phosphate-solubilizing bacteria. Sci. Total Environ..

[16] Xie J., Yan Z., Wang G., Xue W., Li C., Chen X., Chen D. (2021). A bacterium isolated from soil in a karst rocky desertification region has efficient phosphate-solubilizing and plant growth-promoting ability. Front. Microbiol..

[17] Penrose D.M., Glick B.R. (2003). Methods for isolating and characterizing ACC deaminase-containing plant growth-promoting rhizobacteria. Physiol. Plant..

[18] Shin S.H., Lim Y., Lee S.E., Yang N.W., Rhee J.H. (2001). CAS agar diffusion assay for the measurement of siderophores in biological fluids. J. Microbiol. Methods.

[19] Wakarera P.W., Ojola P., Njeru E.M. (2022). Characterization and diversity of native *Azotobacter* spp. isolated from semi-arid agroecosystems of Eastern Kenya. Biol. Lett..

[20] Zhang T., Li T., Zhou Z., Li Z., Zhang S., Wang G., Xu X., Pu Y., Jia Y., Liu X. (2023). Cadmium-resistant phosphate-solubilizing bacteria immobilized on phosphoric acid-ball milling modified biochar enhances soil cadmium passivation and phosphorus bioavailability. Sci. Total Environ..

[21] (2018). Soil Environmental Quality-Risk Control Standard for Soil Contamination of Agricultural Land.

[22] Li H., Zhu H., Li H., Zhang Y., Xu S., Cai S., Sulaiman A., Kuzyakov Y., Rengel Z., Zhang D. (2023). Dynamics of root-microbe interactions governing crop phosphorus acquisition after straw amendment. Soil Biol. Biochem..

[23] Sui Y., Thompson M.L., Shang C. (1999). Fractionation of phosphorus in a Mollisol Amended with biosolids. Soil Sci. Soc. Am. J..

[24] Lu R.K. (2000). Analytical Methods for Soil and Agro-Chemistry.

[25] Liu Y., Lv H., Yang N., Li Y., Liu B., Rensing C., Dai J., Fekih I.B., Wang L., Mazhar S.H. (2019). Roles of root cell wall components and root plaques in regulating elemental uptake in rice subjected to selenite and different speciation of antimony. Environ. Exp. Bot..

[26] Zhao Y., Hu C., Wu Z., Liu X., Cai M., Jia W., Zhao X. (2019). Selenium reduces cadmium accumulation in seed by increasing cadmium retention in root of oilseed rape (*Brassica napus* L.). Environ. Exp. Bot..

[27] Liu N., Tang J., Ye W., Li C., Bao T. (2026). Spatial partitioning and threshold responses of cadmium accumulation and translocation in *Populus* × *euramericana*. Environ. Sci. Eur..

[28] Jiang T., Xu Q., Zhang J., Fu Y., Yuan Q., Zhou T., Xiao C. (2025). *Myrmecridium schulzeri* B-4 promotes the growth of *Bletilla striata* (Thunb.) Reichb. f. through the production of IAA. Ind. Crops Prod..

[29] Chen W., Wu Y.X., Gao Y., Wang B., Shi G., Li Y.T., Xu S., Sheng X.F., Liu L.Z. (2026). Endophyte *Bacillus* sp. RE35 enhances Cd trap in rice roots via root cell wall remodelling during colonisation. Plant Cell Environ..

[30] Wei Y., Zhao Y., Shi M., Cao Z., Lu Q., Yang T., Fan Y., Wei Z. (2018). Effect of organic acids production and bacterial community on the possible mechanism of phosphorus solubilization during composting with enriched phosphate-solubilizing bacteria inoculation. Bioresour. Technol..

[31] Wongcharee S., Kandasamy B., Govindasamy P., Tansomros P., Hongthong S., Sangsida W., Phibanchon S., Chotigawin R., Pahasup-anan T., Pannaracha P. (2025). Pig bone-derived biochar from food industry waste for heavy metal remediation: Sustainable consumption and production. Results Eng..

[32] Zhao J., Gao F., Sun Y., Fang W., Li X., Dai Y. (2021). New use for biochar derived from bovine manure for tetracycline removal. J. Environ. Chem. Eng..

[33] Zhu X., Lv B., Shang X., Wang J., Li M., Yu X. (2019). The immobilization effects on Pb, Cd and Cu by the inoculation of organic phosphorus-degrading bacteria (OPDB) with rapeseed dregs in acidic soil. Geoderma.

[34] An N., Zhang L., Liu Y., Shen S., Li N., Wu Z., Yang J., Han W., Han X. (2022). Biochar application with reduced chemical fertilizers improves soil pore structure and rice productivity. Chemosphere.

[35] Li Z., Liu Z., Wu D., Hu Z. (2024). Enhanced phosphorus availability and cadmium remediation using phosphate-solubilizing bacteria-loaded biochar in contaminated soils. Environ. Technol. Innov..

[36] Zhu X., Wang K., Ma X., Zhang Z., Wang J., Zhang X., Shen B., Si S. (2024). Loading organic phosphorus-degrading bacteria enhanced biochar performance for heavy metals adsorption. Environ. Technol. Innov..

[37] Cui S., Ke Y., Fu Q., Hough R., Zhang Z., Shen Z., An L., Li Y.F. (2022). Optimization preparation of biochar from garden waste and quantitative analysis for Cd^2+^ adsorption mechanism in aqueous solution. Biomass Convers. Biorefin..

[38] Chen Y., Wu X., Lin Z., Teng D., Zhao Y., Chen S., Hu X. (2024). Screening of cadmium resistant bacteria and their growth promotion of *Sorghum bicolor* (L.) Moench under cadmium stress. Ecotoxicol. Environ. Saf..

[39] Qin X.C., Guo S.F., Zhai L.M., Pan J.T., Khoshnevisan B., Wu S.X., Wang H.Y., Yang B., Ji J.H., Liu H.B. (2020). How long-term excessive manure application affects soil phosphorus species and risk of phosphorus loss in fluvo-aquic soil. Environ. Pollut..

[40] Wan W.J., He D.L., Li X., Xing Y.H., Liu S., Ye L.P., Yang Y.Y. (2021). Linking rare and abundant *phoD*-harboring bacteria with ecosystem multifunctionality in subtropical forests: From community diversity to environmental adaptation. Sci. Total Environ..

[41] Vasarevičius S., Paliulienė V. (2025). Immobilization of cadmium, lead, and copper in soil using bacteria: A literature review. Land.

[42] Li Y., Luo S., Fu Y., Tang C., Qin X., Shi D., Lan W., Tang Y., Yu F. (2025). Phosphate-solubilizing bacteria facilitate rhizospheric processes of *Bidens pilosa* L. in the phytoremediation of cadmium-contaminated soil: Link between phosphorus availability and cadmium accumulation. J. Hazard. Mater..

[43] An P., Li X., Zheng Y., Eneji A.E., Inanaga S. (2014). Calcium effects on root cell wall composition and ion contents in two soybean cultivars under salinity stress. Can. J. Plant Sci..

