# Supplementary material for: Efficient Cadmium-Tolerant Phosphate-Solubilizing Bacteria Loaded on Bone-Derived Biochar Effectively Alleviated Cadmium Pollution and Phosphorus Deficiency in Brassica napus L. Plantation
Source: Microorganisms. 2026 Sep 16;14(9):2072. doi: 10.3390/microorganisms14092072 (PMC13609747; doi:10.3390/microorganisms14092072)
Supplement: Supplementary file 1 [file microorganisms-14-02072-s001.zip › microorganisms-4486972-supplementary.pdf]

Supplementary data

Table S1 The mean number of viable functional bacteria loaded on biochar

Biochar carriers	RHB	PBB
Mean number of viable bacteria (10^8 CFU g^{-1})	0.22	5.50

Table S2 Soil main physicochemical properties for pot experiment

pH	TN (%)	TP (mg kg^{-1})	AP (mg kg^{-1})	SOC (mg kg^{-1})
6.17	0.19	427.89	20.60	29.33

Note: TN: Total nitrogen content; TP: Total phosphorus content; AP: available phosphorus content; SOC: Soil organic carbon content
